# Sharing more than just the pastures? Parasitological monitoring of translocated European bison (*Bison bonasus*) and co-grazing cattle in Switzerland

**DOI:** 10.1016/j.ijppaw.2026.101267

**Published:** 2026-07-24

**Authors:** Tobias Heiri, Caroline F. Frey

**Affiliations:** Institute of Parasitology, Department of Infectious Diseases and Pathobiology, Vetsuisse Faculty, University of Bern, Länggassstrasse 122, Bern, 3012, Switzerland

**Keywords:** *Bison bonasus*, Endoparasites, Transmission risk, Translocation, Wildlife-livestock interface

## Abstract

Numbers of European bison (*Bison bonasus*) reintroduction programmes are increasing across Europe. Investigating potential health risks related to translocations is crucial for the long-term success of such programmes. Parasitological analyses play a central role in monitoring the health status and assessing disease transmission risks during the translocation of European bison herds. This study monitored the endoparasite fauna of both a newly relocated European bison herd (n = 7 animals), and of the neighbouring suckler cow herd (n = 25 animals), over the course of one year. The aim of the study was to compare the parasite populations of these two closely related ruminant species kept under similar management conditions, and to assess the risk of parasite transmission by shared grazing areas. From April 2023 to March 2024, faecal samples from both herds were analysed monthly, using direct detection methods such as sedimentation, combined sedimentation-flotation, Baermann funnel, McMaster, and copro-antigen tests (total n = 72 samples). A total of seven helminth groups and four protozoan genera were thereby identified, with a richer parasite diversity in the bison samples. Eggs of *Trichuris* spp., *Nematodirus* spp., *Moniezia* spp., and cysts of *Giardia* sp. were exclusively shed by the European bison. On the other hand, cysts of *Buxtonella* sp. were only found in cattle samples. Paramphistomidae eggs and *Cryptosporidium* spp. were significantly more frequently detected in cattle samples. Both, the suckler cow and the European bison herd, showed constant infection with gastrointestinal strongylids, but the faecal egg counts were tenfold higher in the bison. Infections with *Eimeria* spp., *Strongyloides* spp., and *Dicrocoelium dendriticum* were detected in both species, and no lungworm larvae were detected in either species. Further investigations should include identification of the parasites to species level, and the parasitological monitoring in the two herds should be continued to assess if the observed differences persist or disappear over time.

## Highlights

• Relocated European bison showed higher parasite diversity than local suckler cattle.
• Gastrointestinal strongyle egg shedding was 10 times higher in European bison compared to the local suckler cattle herd.
• 7 of 11 parasite groups were found in bison and cattle, suggesting a potential wildlife–livestock parasite transmission risk.
• This study provides baseline data for Swiss European bison reintroduction programs.

## Graphical abstract

**Figure:**
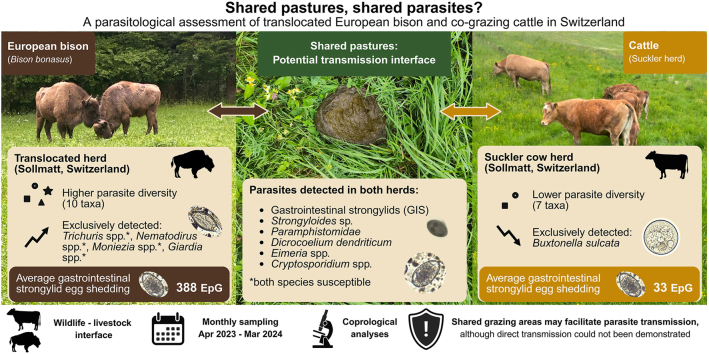

## 1 Introduction

The European bison (*Bison bonasus*) looks back on a turbulent history with a remarkable comeback in the last century. Following centuries of population decline due to overhunting and habitat loss, the lowland wisent (*Bison bonasus*) was eradicated in the wild by 1919. The same fate befell the Caucasian wisent (*Bison bonasus caucasicus*) in 1927. Extinction was only prevented by a small number of individuals held in captivity (Krasińska and Krasiński, 2013). Thanks to breeding and reintroduction programmes in several countries, particularly Poland, the population has recovered from just twelve founding individuals to over 9500 animals, with the wild population currently exceeding 6200 individuals (IUCN, 2020; Raczynski & Bolbot, 2023). Despite the success of European bison conservation programmes, translocation of wild animals always carries the risk of spreading pathogens. The close phylogenetic relationship between European bison and domestic cattle (*Bos taurus*), as well as other wild ruminants, presents a significant potential for cross-species disease transmission (Gortázar et al., 2007). The European bison is highly susceptible to parasitic infections, likely as a result of inherited immunodeficiency associated with the species’ narrow genetic bottleneck or of restricted habitat range and thus higher exposure to infective parasite stages (Radwan et al., 2010; Krasińska and Krasiński, 2013; Kołodziej-Sobocińska et al., 2018). Consequently, parasite transmission represents a major concern in European bison reintroduction programmes. Moreover, only four of the more than 88 parasite species recorded in European bison are considered host-specific (*Trypanosoma wrublewskii*, *Demodex bisonianus*, *Demodex bialoviensis* sp. nov., and *Bisonicola sedecimdecembrii)*, further underscoring the importance of systematic parasite monitoring (Karbowiak et al., 2014a; Izdebska et al., 2022).

A notable example highlighting the risk for parasite transmission is the introduction of *Ashworthius sidemi*, a pathogenic blood-feeding nematode, into the red deer population of the Czech Republic through translocated European bison (Vadlejch et al., 2017). This case demonstrates the potential for reintroduction programmes to facilitate parasite transmission across ruminant species. Parasitological screening should therefore be regarded as an essential component of health assessments in European bison translocation and reintroduction programmes (Larska and Krzysiak, 2019; Buchmann et al., 2022).

At present, Switzerland does not have free-ranging European bison populations. In autumn 2022, however, a European bison herd has been introduced to a fenced area on a farm called “Sollmatt” located in the Jura Mountains of the canton of Solothurn. The project assesses the impact of free-ranging European bison on their habitat in Switzerland with the long-term goal of the release of the herd into the wild (Verein Wisent Thal, 2020). One aspect of the ongoing research involves the continued agricultural use of pastureland within the enclosure, including rotational grazing by a local suckler cow herd. While there is no direct contact between the bison and the cattle, the use of shared pastures may still pose a risk for parasite transmission. The aim of this study was to characterise the parasitic fauna of the European bison herd following a soft release and being left untreated, and to quantitatively document the seasonal shedding patterns of gastrointestinal strongylid eggs (GIS eggs) by monthly analysing faecal samples over the course of a year (April 2023 – March 2024). The findings were then compared and interpreted with the results of the simultaneously sampled local cattle herd, focusing on the risk of possible parasite transmission. The data obtained from this study could serve as a reference baseline for future studies on the parasitological status of European bison in rewilding programmes and eventually in total freedom in Switzerland.

## 2 Materials and methods

### 2.1 Study area

**Fig. 1:**
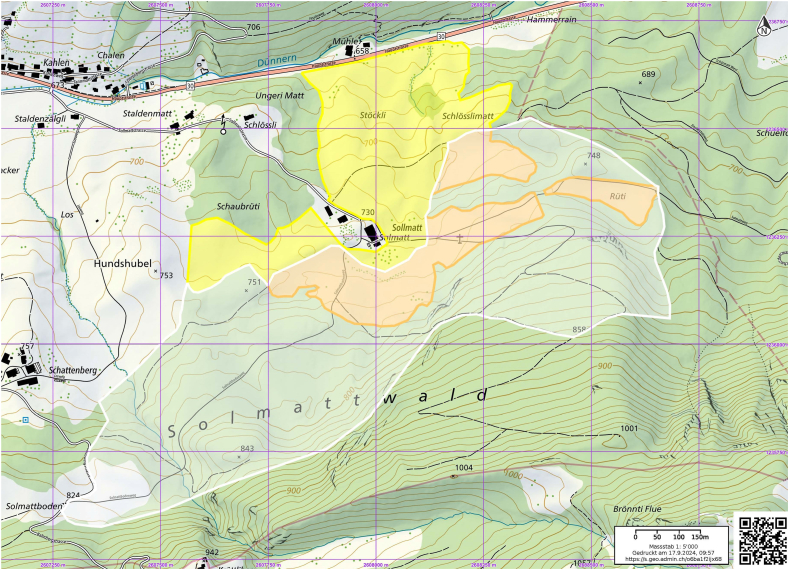
Map of the study area with shared grazing areas marked orange, the fenced European bison enclosure in white and additional cattle pastures in yellow.

The European bison enclosure (Fig. 1, white and orange areas) and the adjacent cattle grazing areas (Fig. 1, yellow area) were located on the Sollmatt site (47.276° N, 7.544° E; 730 – 858 m a.s.l.) in Welschenrohr, Jura Mountains, Switzerland. The bison enclosure covered a total area of 51 ha, consisting of 10 ha of pasture and grassland and 41 ha of forest (Verein Wisent Thal, 2020). Grazing areas within the bison enclosure (Fig. 1, orange areas) were used by the local suckler cow herd from June 9th to 23rd, 2023, and October 7th to November 25th, 2023. During these periods, the bison were excluded from the orange areas. Direct contact between the two herds was prevented by two temporarily installed electric fences, creating a 5-m-wide buffer zone. The fence structures allowed the passage of other local wildlife species, including red deer (*Cervus elaphus*), roe deer (*Capreolus*), chamois (*Rupicapra*), wild boar (*Sus scrofa*), lynx (*Lynx*), and wolf (*Canis lupus*), among others (Amt für Wald, Jagd und Fischerei Kanton Amt für WaldJagd und Fischerei Kanton Solothurn, 2024).

### 2.2 Study population

#### 2.2.1 European bison herd

At the beginning of the sampling period, the European bison herd consisted of five founder animals introduced to the enclosure on September 15th, 2022 (Table 1). All animals were bred in two zoos in Switzerland, the four female animals in one zoo, while the bull came from another zoo (Table 1). Prior to translocation, the animals were routinely screened for parasitic infections at their breeding sites by pooled samples, revealing infection with coccidia, GIS and *Trichuris* sp. Except for one European bison cow and her calf, all founder animals were dewormed with Ivermectin (Virbamec®, Virbac AG, Glattbrugg, Switzerland. 0.2 mg/kg BW, *sub cutem*) three weeks (bull and a cow) or one week (1 cow), respectively, before translocation (Table 1). Two cows were radio collared. The effect of deworming was not controlled for. No more antiparasitic treatments were administered after translocation. In the first half of July 2023, *i.e.* during the sampling period, two calves were born, increasing the herd size to seven individuals. Over the course of the sampling period, no clinical signs of illness or parasite infection were observed. In addition to natural forage available within the enclosure, the herd was supplemented with hay from the adjacent suckler cow operation during the winter months. To facilitate herd management, Granovit Browser 3699 (Granovit AG, Kaiseraugst, Switzerland) was used as an attractant feed, but no minerals were supplemented.

**Table 1:** European bison herd Sollmatt.

Sex	Date of birth	Origin	Deworming status
female	27 Aug. 2017	Wildnispark Zürich, Langenberg	Fenbendazole^a^ 27 Dec. 2021
female	17 June 2018	Wildnispark Zürich, Langenberg	Ivermectin^b^ 24 Aug. 2022
female	03 May 2019	Wildnispark Zürich, Langenberg	Ivermectin 08 Sept. 2022
male	05 June 2019	Wildpark Bruderhaus, Winterthur	Ivermectin 24 Aug. 2022
female	12 July 2022	Wildnispark Zürich, Langenberg	no treatment
female	04 July 2023	Sollmatt, Welschenrohr	no treatment
male	14 July 2023	Sollmatt, Welschenrohr	no treatment

a Panacur® 10%, MSD Animal Health, Lucerne, Switzerland. 7.5 mg/kg BW, *per os*. Acts against gastrointestinal nematodes and *Moniezia* spp., partially active against *Dicrocoelium dendriticum* and *Fasciola hepatica*.

b Virbamec®, Virbac AG, Glattbrugg, Switzerland. 0.2 mg/kg BW, *sub cutem*. Acts against nematodes and ectoparasites.

#### 2.2.2 Cattle herd

Throughout the sampling period, the suckler cow herd consisted of one bull and an average of twelve cows with calves, totalling approximately 25 individuals. The herd was of mixed breed, including Limousin, Simmental, and Raethian Grey cattle. Seasonal calving occurred between January and April. No new animals were introduced during the study period, and no antiparasitic treatments were administered during the sampling period or in previous years. Records from routine meat inspection indicated the occurrence of liver fluke infections among slaughtered animals. However, no information on fluke species identification was available. No clinical signs of illness or parasite infection were observed over the sampling period. The herd's diet varied seasonally and included pasture, home-produced silage and hay. UFA 994 mineral feed (UFA AG, Herzogenbuchsee, Switzerland) was used as a dietary supplement.

### 2.3 Sample collection

Sampling was carried out monthly from April 2023 to March 2024. Each time, three faecal samples, each in about 5 m distance to the next one, were collected from the European bison herd and the suckler cow herd, respectively, totalling in 72 faecal samples. Sampling of the bison herd was consistently conducted in the morning hours to ensure the collection of fresh, still warm, faeces from the animals’ overnight resting areas. The location of the bison herd was determined using a Telonics TR-8 telemetry device (Telonics Inc., Mesa, Arizona, USA). As a minimum distance of 50 m had to be maintained from the bison herd, direct observation of defecation events was not feasible, and samples could not be attributed to specific individuals. Sampling within the cattle herd followed the same protocol. All samples were individually analysed on the day of collection.

### 2.4 Analysis

**Fig. 2:**
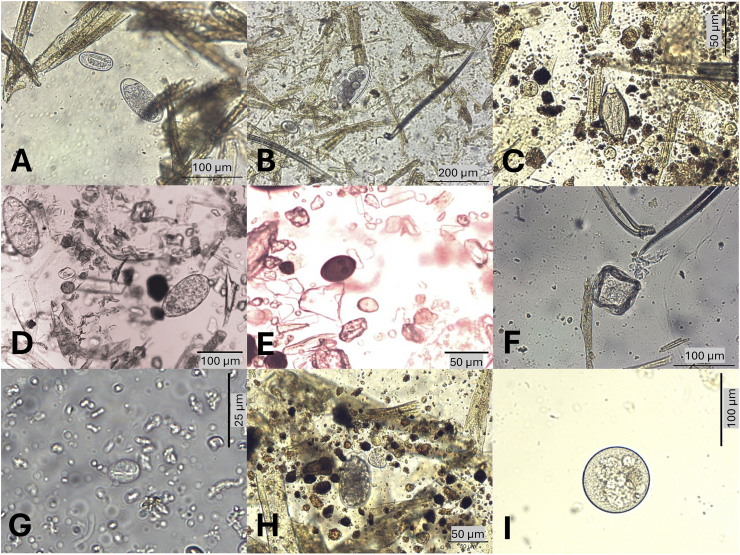
A: *Strongyloides* sp. egg (left) detected in European bison sample next to a GIS egg (right). B: *Nematodirus* spp. egg detected in European bison sample. C: *Trichuris* spp. egg detected in European bison sample. D: Paramphistomidae eggs detected in European bison sample. E: *Dicrocoelium* sp. egg detected in cattle sample. F: *Moniezia* spp. egg detected in European bison sample. G: *Giardia* sp. cyst detected in European bison sample. H: *Eimeria* spp. oocyst (right) and GIS egg (left) detected in European bison sample. I: *Buxtonella* sp. cyst detected in cattle sample.

All faecal samples (n = 72) were examined with classical coprological methods. For detection of cestode and nematode eggs, as well as coccidia oocysts, a combined sedimentation-flotation method with a 44% zinc chloride solution (specific gravity 1.45) starting from 20g faeces was used (Deplazes et al., 2021). For trematode eggs and *Buxtonella* cysts, the sedimentation technique described by Deplazes et al. (2021) was applied, also using 20g faeces. A McMaster method using 3 g of faeces and with a 50 Eggs per Gramm faeces (EpG) detection limit was performed to quantify the excretion of GIS eggs (Taylor et al., 2007). Baermann-Wetzel technique starting from 25 g faeces was used for the detection of lungworm larvae (Deplazes et al., 2021). Parasite identification followed morphological criteria according to Deplazes et al. (2021), which allowed for identification of parasite groups or genera, but not for identification to species-level (Fig. 2). Additionally, a commercial copro-antigen test (FASTest® CRYPTO-GIARDIA Strip ad us. vet.; Megacor GmbH, Germany) was used to detect *Cryptosporidium* spp. and *Giardia duodenalis*. In the case of positive results in the antigen test, additional Ziehl-Neelsen staining or Sodium acetate-Acetic acid-Formalin concentration method (SAFC; Deplazes et al., 2021) were conducted. The antigen rapid test was not available for the first sampling, thus Ziehl-Neelsen staining and SAFC were used to detect *Cryptosporidium* spp., and *Giardia* sp., respectively.

Statistical analyses were carried out to evaluate group differences in parasite prevalence between the two study populations. The proportions of positive faecal samples, along with their 95% confidence intervals (95% CI), were calculated using the Wilson method. Fisher's exact test was employed to assess the significance of differences in the proportion of positive samples between cattle and bison herd. For quantitative comparison of GIS egg count (expressed as eggs per gram of faeces, EpG), Wilcoxon signed-rank test was applied. All statistical tests were two-tailed, and p-values ≤0.05 were considered statistically significant. Analyses were conducted in R (version 4.4.1) using the ‘binom’ package (version 1.1.1.1) and the ‘tidyr’ package (version 1.3.1). Pictures and measurements were conducted using a Nikon Eclipse Ci microscope, a calibrated Nikon Camera (model DFK 23UP031) and NIS software (Nikon).

## 3 Results

### 3.1 Detected parasites

Over the study period, a total of seven helminth groups and four genera of protozoa were identified (Table 2, Fig. 2). The most frequently detected parasite stages were eggs of GIS being found in all but one sample (Table 3). Additionally, eggs of *Nematodirus* spp. were exclusively detected in two samples of the bison herd. Statistically significant differences (p < 0.05) were observed for the nematode genus *Trichuris* spp., which was detected exclusively in 19% of the bison herd samples (Table 2). *Strongyloides* sp. were found to a similar degree in both species (Table 2, Fig. 2). Two genera of trematodes were identified by sedimentation, Paramphistomidae and *Dicrocoelium dendriticum*. Paramphistomidae eggs were detected throughout the study period, except in the October samplings of the bison herd. Further, the cattle herd tested significantly more often positive for Paramphistomidae (p < 0.05, Table 2). One bison sample tested positive for *D*. *dendriticum* and four positive results were found in samples of the cattle herd. *Moniezia* spp. was exclusively detected in six bison samples (p < 0.05). Protozoa were frequently detected. *Eimeria* spp. were found in most samples in both groups. The single positive antigen test result for *Giardia duodenalis* in a bison sample was confirmed by cysts in the SAFC. On the other hand, positive antigen test results for *Cryptosporidium* spp. could not be confirmed by Ziehl-Neelsen staining. Based on the antigen test results, *Cryptosporidium* spp. was significantly more often detected in the cattle herd (p = 0.002). *Buxtonella* sp. was identified exclusively in 58% of the cattle samples (p < 0.001).

**Table 2:** Overview of detected parasite eggs and (oo)cysts with proportions of positive samples, confidence interval of prevalences and p-values (sig. values in bold).

Taxa	Proportion of positive samples		95% Confidence Interval		p-value
	Europ. bison	Cattle	Europ. bison	Cattle	
*Strongyloides* sp.	19% (7/36)	11% (4/36)	10 – 35%	4 - 25%	0.514
Gastrointestinal strongylids	100% (36/36)	97% (35/36)	90 - 100%	86 - 100%	1
*Nematodirus* spp.	6% (2/36)	0% (0/36)	2 - 18%	0 - 10%	0.493
*Trichuris* spp.	19% (7/36)	0% (0/36)	10 – 35%	0 - 10%	**0.011**
Paramphistomidae.	47% (17/36)	75% (27/36)	32 - 63%	59 - 86%	**0.029**
*Dicrocoelium dendriticum*	3% (1/36)	11% (4/36)	0 - 14%	4 - 25%	1
*Moniezia* spp.	17% (6/36)	0% (0/36)	8 - 32%	0 - 10%	**0.025**
*Giardia duodenalis*	3% (1/36)	0% (0/36)	0 - 14%	0 - 10%	1
*Eimeria* spp.	75% (27/36)	67% (24/36)	59 - 86%	50 - 80%	0.605
*Cryptosporidium* spp.	14% (5/36)	50% (18/36)	6 - 29%	34 - 66%	**0.002**
*Buxtonella sulcata*	0% (0/36)	58% (21/36)	0 – 10%	42 – 73%	**< 0.001**

**Table 3:**
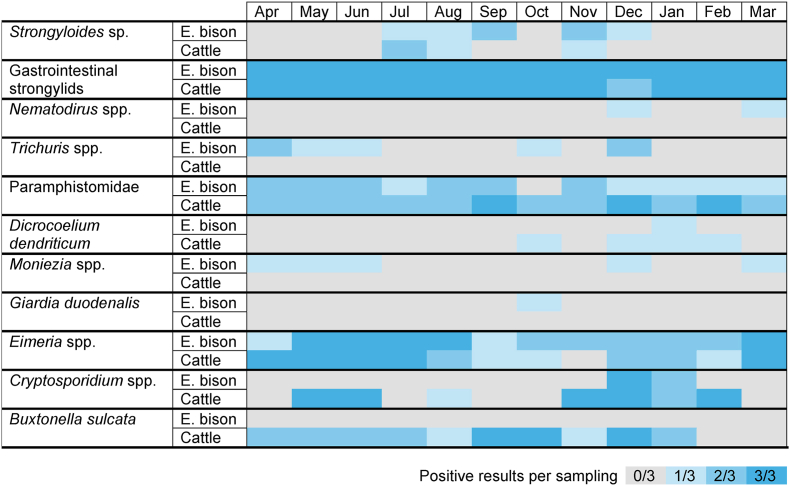
Distribution of positively tested samples throughout the study period (April 2023 – March 2024).

### 3.2 Gastrointestinal strongylid egg excretion

**Fig. 3:**
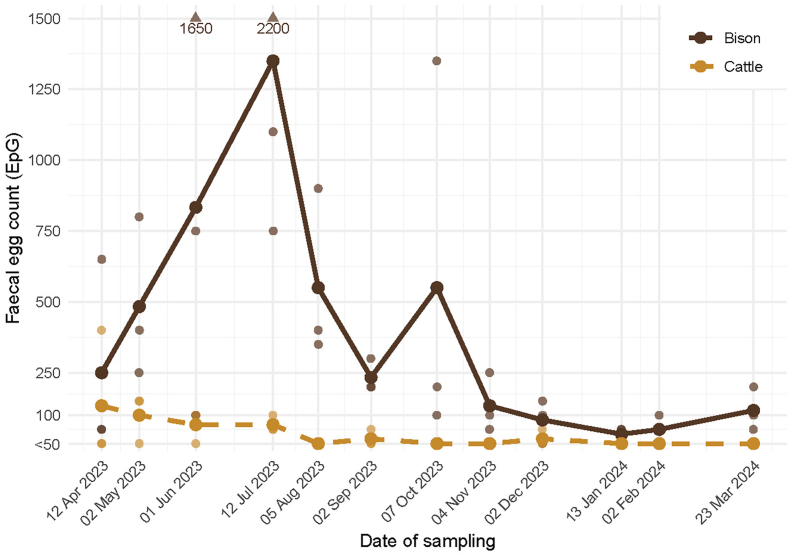
Temporal dynamics of gastrointestinal strongylids (GIS) egg shedding over the study period, showing average eggs per gram faeces (EPG) and individual sample values visualized as dots and crosses.

The quantitative assessment of GIS egg excretion showed markedly higher levels and a more dynamic pattern in the bison herd compared to the cattle herd (Fig. 3). In July 2023, at the time the two calves were born, a peak in EpG was observed in the bison samples, with an average EpG count of 1′350 EpG, and a median of 1′100 EpG (Fig. 3). From July to January, EpG levels in the bison herd fell to lower values, approaching cattle levels in winter (Fig. 3). Towards spring 2024, EpG values in bison samples began to increase again. The EpG values of the cattle herd were highest at the start of the study period in April 2023, due to a peak value of 400 EpG in one sample. Subsequently, low egg shedding was observed in the cattle herd (Fig. 3). Across the entire study period, the European bison herd showed an average GIS egg shedding of 388 EpG, with a median of 200 EpG. In the cattle herd, the average egg excretion was 33 EpG. Throughout the study period, European bison showed significantly higher mean EpG counts than cattle (paired Wilcoxon signed-rank test; p < 0.01).

## 4 Discussion and conclusions

The results obtained in this study confirm a fundamental overlap of the parasite faunas of European bison and domestic cattle. Seven out of eleven detected parasite groups were present in samples from both species. Four additional parasite genera identified exclusively in European bison samples (*Nematodirus* spp., *Trichuris* spp., *Moniezia* spp., *Giardia* sp.) are further recognized to be part of the parasitological fauna of domestic cattle, too (Deplazes et al., 2021).

*Strongyloides* spp. was detected at comparable rates in bison (95% CI: 10–35%) and the cattle herd (95% CI: 4–25%). To date, only Krzysiak et al. (2020) have reported *Strongyloides* spp. infection in European bison, whereby no species-level identification was performed. Based on morphological features of the detected eggs (Fig. 2), we suggest it to be *S. papillosus,* as this species is commonly found in central Europe in other wild ruminants, as well as in cattle (Deplazes et al., 2021).

No species-level identification of GIS was carried out in this study. Previous molecular studies most frequently found *Haemonchus contortus*, *Ostertagia ostertagi*, and *Cooperia oncophora* in the GIS population of European bison (Pyziel et al., 2018; Herskind et al., 2023). As the two latter species are commonly found in domestic cattle, it is likely that GIS detection in both herds carries the risk for species transmission (Karbowiak et al., 2014b).

*Trichuris* sp. was exclusively detected in 19% (95% CI: 10–35%) of European bison samples, although a low prevalence (0–10%) in the cattle herd cannot be ruled out. The bison reportedly harboured *Trichuris* sp. prior to translocation, and as no post-treatment control after ivermectin administration was performed, and one female bison and her calf were left untreated, the relocation of this parasite along with the European bison herd onto the Sollmatt cannot be ruled out. The only whipworm species reported in European bison is *Trichuris ovis* (Karbowiak et al., 2014a; Kołodziej-Sobocińska et al., 2016; Gałązka et al., 2023), and this species is also part of the parasitic fauna of domestic cattle, considered a generalist in regard to different ruminant host species (Winter et al., 2018; Deplazes et al., 2021). Thus, cross-species transmission of *Trichuris* sp. via shared pastures might be a likely scenario.

Paramphistomidae were significantly more often detected in samples of the cattle herd (p = 0.029). The CI show a roughly two times higher infection rate compared to the European bison herd, aligning with the increasing rumen fluke findings in central European cow populations (Huson et al., 2017). With *Paramphistomum cervi* at least one rumen fluke species is known to parasite both ruminants (Karbowiak et al., 2014a; Hecker et al., 2024). Buchmann et al. (2022) further suggests *Calicophoron daubneyi* (a rumen fluke frequently found in cows) to be part of the European bison's parasite fauna. Thus, the finding of rumen flukes poses a possible parasite transmission risk.

Notably, the common liver fluke (*Fasciola hepatica*), considered pathogenic in European bison and sharing the same intermediate host as *C. daubneyi* (Krzysiak et al., 2014; Jones et al., 2017), has not been detected in our study. Therefore, we assume that the liver fluke infection in cattle reported at the slaughterhouse was likely to be caused by the small liver fluke, *D. dendriticum* which was found in this study.

*Moniezia* spp. were exclusively found in six faecal samples of the European bison herd. *Moniezia benedeni* and *M. expansa* are the two relevant *Moniezia* species in European cattle (Deplazes et al., 2021). Both species are further considered to be part of the European bison's parasite fauna (Karbowiak et al., 2014a; Buchmann et al., 2022). Despite two documented cases of deadly *Moniezia* infection, Buchmann et al. (2022) suggest the overall impact of *Moniezia* spp. on bison health to be minor. Infection dynamics of *Moniezia* spp. at the Sollmatt site remain inconclusive. Parasitological examinations conducted prior to the European bison herd relocation did not report *Moniezia* spp. infection. However, positive samples were sporadically found in the European bison herd throughout the study period. The lack of detection in the cattle herd likely reflects a low prevalence (95% CI = 0–10%) in this population, but does not rule it out, leaving the origin of *Moniezia* spp. detected in the European bison herd unresolved.

Three of the four identified protozoan genera have been described in both cattle and European bison (Karbowiak et al., 2014a; Deplazes et al., 2021). *Buxtonella sulcata* has been found exclusively in samples from the cattle herd. This finding aligns with Karbowiak et al. (2014a), who did not describe *B. sulcata* as part of the European bison's parasite fauna.

The single detection of *Giardia* antigen and cysts in one bison sample suggests low prevalence in the European bison herd, and apparent freedom in the cattle herd, respectively. Nonetheless *Giardia* sp. can be found in both species (Karbowiak et al., 2014a). *Eimeria* spp. were regularly detected in both herds, but are generally very host specific, thus may be different species in the two host populations (Table 2). In contrast, the difference in *Cryptosporidium* spp. occurrence was statistically significant (p = 0.002). Half of the faecal samples from the cattle herd tested positive for *Cryptosporidium* antigen (CI: 34–66%), while only five samples from the European bison herd tested positive (CI: 6–30%). The first positive finding in a European bison sample was detected in December 2023, more than one year after translocation. Hatam-Nahavandi et al. (2019) state that due to its resilience and a generally higher prevalence of *Cryptosporidium* spp. in domestic ruminants, they may act as a potential parasite reservoir for wild ruminants. This is particularly noteworthy, as Edwards et al. (2026) reported that European bison host *Cryptosporidium* species typically associated with cattle, namely *C. parvum* and *C. bovis*. To interpret the delayed positive findings in the European bison herd as evidence for parasite transmission from cattle into the European bison herd remains speculative, since the European bison herd has not been screened for *Cryptosporidium* spp. before relocation. Nevertheless, given the shared grazing areas, a potential risk of transmission for *Cryptosporidium* spp. (as well as *G. duodenalis*) must be assumed.

The shedding of GIS eggs was quantified over the course of the study period (Fig. 2), and a highly significant difference (p < 0.01) was found in average EpG count between the two herds. Faecal samples from the bison herd contained over ten times more EpG (388 EpG) compared to samples from the cattle herd (33 EpG). This low shedding pattern has been previously reported by Wills et al. (2020) in suckler cow herds. In contrast, the European bison samples reached high EpG values (up to 2200 EpG). The EpG count of the European bison test herd in this study aligns with previous observations. Gałązka et al. (2022) reported in a study with 166 samples of Polish European bison a median of 150 EpG, an average of 239 EpG and a range of 50–2950 EpG. Gałązka et al. (2023) further reported an average egg count of 386 EpG, comparable to the results of this study. The median value of 200 EpG matches that of a bison herd relocated to Lille Vildmose (Herskind et al., 2023). The hypothesis that European bison might have a hereditarily increased susceptibility to gastrointestinal parasites is frequently raised (Kołodziej-Sobocińska et al., 2018). However, testing to what extent a genetic bottleneck affects the immunity of a species is inherently challenging (Radwan et al., 2010). The restricted movement of the bison in the enclosure also might lead to the accumulation of infective parasite stages on the pastures and thus higher parasite burden. Free-roaming bison would typically migrate over long distances, thus possibly avoiding highly contaminated areas. Elevated parasite burdens after relocations can also reflect insufficient immunity development in captivity, for example due to regular deworming or lack of exposure to specific parasites (Mathews et al., 2006; Kołodziej-Sobocińska et al., 2018). This aspect also applies to the test herd on the Sollmatt. In this context Kołodziej-Sobocińska et al. (2018) showed significantly higher infections with *A*. *sidemi* in reintroduced European bison compared to established wild populations. Beyond generally higher EpG levels, the GIS egg shedding of the bison herd showed a major peak in July. This observation contrasts sharply with patterns reported in Polish European bison populations and resembles the well-known epidemiological trend of GIS egg shedding in (young) cattle (Deplazes et al., 2021). The Polish European bison population in Białowieża Forest shows significantly higher GIS EpG values in winter (December–March) compared to summer. Possible factors for this seasonal effect include high animal density at winter feeding stations, the presence of cold-resistant infectious larval stages of certain trichostrongylid species (e.g., *Trichostrongylus colubriformis*) in combination with reduced body condition in winter, leading to increased susceptibility to pathogens (Kołodziej-Sobocińska et al., 2016). In contrast, the small herd size (five to seven animals) and the winter-feeding strategy using hay racks on the Sollmatt site may be factors contributing to a lower infection pressure over the winter period in the present herd. The sharp increase in egg shedding up to midsummer might be due to the discussed weak immunity development against GIS parasites in captivity, likely influenced by regular deworming before translocation to the Sollmatt, or by first-time contact with unfamiliar GIS species after translocation. The high values in July might also be explained by periparturient egg rise, a phenomenon well documented in sheep, where parasitic burden and egg shedding increase around lambing or calving (Höglund et al., 2021). While this phenomenon has not been directly described in European bison, the observation by Pyziel et al. (2011) of elevated coccidian oocyst shedding in female European bison during the calving period indicates a similar effect might occur in European bison. However, further and more targeted investigations are needed to prove this hypothesis.

From a conservation perspective, the parasite findings do not indicate an immediate parasite-mediated threat to the health of the Sollmatt bison herd during the observation period. Neither lungworm larvae nor *F*. *hepatica* eggs were detected in the examined samples. Nevertheless, both parasite groups are relevant to European bison health. *Dictyocaulus viviparus* has been associated with pneumonia, emphysema, and other respiratory lesions in European bison, and severe infection has caused fatal verminous pneumonia in a recently reintroduced individual (Krzysiak et al., 2014; Cârstolovean et al., 2024). Similarly, *F. hepatica* has been associated with hepatitis and cirrhosis in European bison and is therefore considered a parasite of pathological significance (Krzysiak et al., 2014). The presence of *A. sidemi* in the Sollmatt herd appears unlikely because the animals originated from a zoological setting, were routinely screened for parasitic infections, and were treated with ivermectin shortly before translocation. However, this interpretation remains speculative, as the strongylid eggs detected in this study cannot be assigned to *A. sidemi*, *H*. *contortus,* or other gastrointestinal strongylid species by flotation alone. Species-level identification would require additional methods, such as larval culture followed by molecular identification or species-specific PCR (Reslova et al., 2021; Gałązka et al., 2024). This is relevant because both *A. sidemi* and *H. contortus* are blood-feeding nematodes with the potential to cause anaemia and severe disease at high infection intensities (Kołodziej-Sobocińska et al., 2016; Skorpikova et al., 2020). Although GIS egg shedding was significantly higher in the bison herd than in the cattle herd, the mean value observed in bison (388 EpG) was comparable to values reported in other European bison populations and together with the absence of observed clinical signs in the bison herd, these findings suggest that the animals were coping with the detected parasite burden under the conditions of the present study, although subclinical effects cannot be excluded. Regarding parasite burden, the same concept applies to the other detected parasites. For example, *Moniezia* spp. was detected sporadically and is generally considered to have a minor impact on bison health, but fatal infections have been documented in some cases (Buchmann et al., 2022). Thus, the present results indicate a potential parasite-transmission interface between bison and cattle, but they do not establish an immediate clinical threat. Continued surveillance with species-resolved diagnostics would nevertheless be warranted, particularly for *A. sidemi, H. contortus, F. hepatica*, and *D. viviparus* during future translocation and reintroduction efforts.

### 4.1 Limitations

The conducted study is subject to numerous limitations. First, the sensitivity of coprological examinations is inherently limited, and false-negative results can occur, for example, due to inconsistent egg shedding. Additionally, the statistical power of the analysis is constrained by small sample sizes. Therefore, negative findings in one herd do not provide definitive proof of the absence of a given parasite genus in that herd. The most significant limitation is insufficient species-level identification, limiting the interpretive strength of the study's findings. As a result, the assessment of transmission risk is largely based on supporting literature with unclear applicability on the local parasite fauna in this study. It must further be emphasized that a potential influence of other wild ruminant species present in the study area on the local parasite fauna was not assessed. Finally, the faecal samples could not be attributed to individual animals, thus repeated sampling of the same animal was not entirely excluded, although we did our best to avoid this.

### 4.2 Conclusion

The study highlights the similarity of the parasite faunas of two similarly managed local populations of European bison and domestic cattle, respectively. The parasite fauna in the European bison was more diverse and egg shedding levels of GIS were higher than in the cattle herd. For all detected parasites except for *Buxtonella* sp., the use of shared pastures holds a potential risk for cross-species parasite transmission. However, transmission of individual parasite genera could not be directly proven within this study. Moreover, the results should serve as a baseline for future parasitological research in Swiss European bison herds and thereby contribute to a deeper understanding of the dynamics and the evolution of the European bison's parasite fauna following relocation and reintroduction.

## Disclaimer and conflicts of interest

During the preparation of this work the authors used ChatGPT (OpenAI) to improve language and grammar of the manuscript. The authors reviewed and edited the content as needed and take full responsibility for the content of the publication.

## Funding

The present study was supported by internal funds from the Institute of Parasitology, Vetsuisse Faculty, University of Bern.

## CRediT authorship contribution statement

**Tobias Heiri:** Conceptualization, Data curation, Formal analysis, Investigation, Methodology, Visualization, Writing – original draft. **Caroline F. Frey:** Conceptualization, Funding acquisition, Supervision, Writing – review & editing.

## Declaration of competing interest

The authors declare that they have no known competing financial interests or personal relationships that could have appeared to influence the work reported in this paper.
